# Toxicological testing of allogeneic secretome derived from peripheral mononuclear cells (APOSEC): a novel cell-free therapeutic agent in skin disease

**DOI:** 10.1038/s41598-019-42057-5

**Published:** 2019-04-03

**Authors:** Silvio Wuschko, Alfred Gugerell, Monika Chabicovsky, Helmut Hofbauer, Maria Laggner, Michael Erb, Tobias Ostler, Anja Peterbauer, Susanne Suessner, Svitlana Demyanets, Jost Leuschner, Bernhard Moser, Michael Mildner, Hendrik J. Ankersmit

**Affiliations:** 1 Drug and Chemical Safety Research & Toxicology, Consultant, Alland, Austria; 2 Division of Thoracic Surgery, Medical University of Vienna, Vienna, Austria; 3 Department of Cardiology, Department of Internal Medicine II, Medical University of Vienna, Vienna, Austria; 4 MC Toxicology Consulting GmbH, Vienna, Austria; 5 Synlab, Birsfelden, Switzerland; 6 Red Cross Blood Transfusion Service of Upper Austria, Linz, Austria; 7 Department of Laboratory Medicine, Medical University of Vienna, Vienna, Austria; 8 LPT - Laboratory of Pharmacology and Toxicology GmbH & Co KG, Hamburg, Germany; 9 Research Division of Biology and Pathobiology of the Skin, Department of Dermatology, Medical University of Vienna, Vienna, Austria; 10 FFG Projects “APOSEC” 852748 and 862068, Medical University Vienna, Vienna, Austria

## Abstract

A cell-free approach using secretomes derived from stem cells or peripheral blood mononuclear cells is an active area of regenerative medicine that holds promise for therapies. Regulatory authorities classify these secretomes as biological medicinal products, and non- clinical safety assessment thus falls under the scope of ICH S6. A secretome of stressed peripheral blood mononuclear cells (APOSEC) was successfully tested in a toxicology program, supporting clinical use of the new drug candidate. Here, to allow for topical, dermal treatment of patients with diabetic foot ulcer, several non-clinical safety studies were performed. Acute toxicity (single dose) and neuropharmacological screening were tested intravenously in a rat model. Risk for skin sensitisation was tested in mice. A 4-week intravenous toxicity study in mice and a 4-week subcutaneous toxicity study in minipigs were conducted to cover the clinical setting and application in a rodent and a non-rodent model. Acute and repeated-dose toxicity studies show that APOSEC administered intravenously and subcutaneously does not involve major toxicities or signs of local intolerance at levels above the intended total human maximal dose of 3.3 U/kg/treatment, 200 U/wound/treatment, and 100 U/cm^2^/treatment. The non-clinical data support the safe topical use of APOSEC in skin diseases related to deficient wound healing.

## Introduction

**Figure 1:**
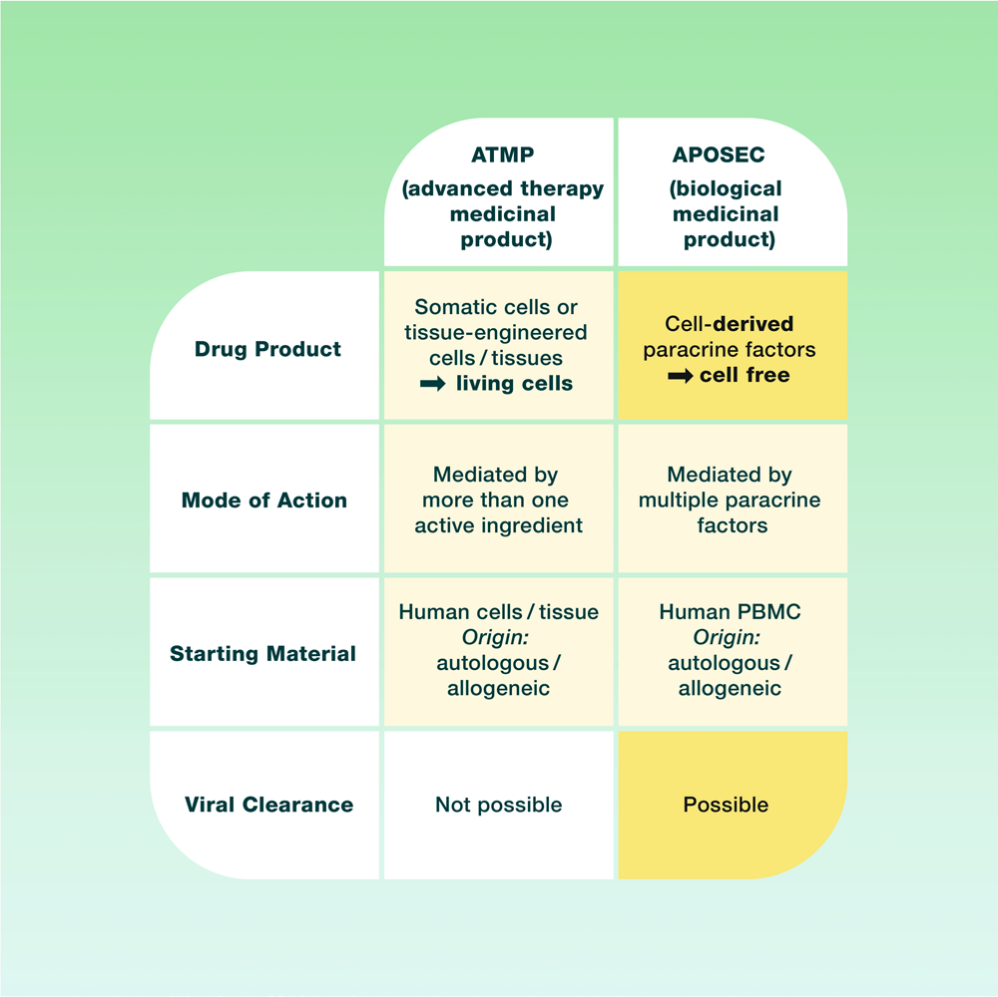
Advantages and drawbacks of ATMP and cell-derived (secretome) biological medicinal products.

The efficacy of stem cell–based therapies depends on soluble factors secreted by the transplanted cells^1–3^. Recently, the secretome of stem cell cultures was identified as a key component for accelerating cutaneous wound healing without cellular compartments^4,5^. The idea of using conditioned medium as a therapeutic agent originated from stem cell–based therapy for myocardial infarction. Studies show that the regenerative therapeutic effects seen after administration of stem cells in acute myocardial infarction are mediated via paracrine signalling rather than by direct cellular interactions^1,6^. By 2008, it was reported that conditioned culture media derived from mesenchymal stem cells and peripheral blood mononuclear cells (PBMCs) are rich in angiogenic and chemotactic factors. Moreover, *in vitro* assays demonstrated that these conditioned media could augment endothelial cell proliferation *in vitro*^7,8^. In contrast to stem cells, PBMCs are easily obtainable and a waste product in blood donation centres all over the world. Therefore, a cell-free product derived from PBMCs instead if stem cells has some advantages compared to advanced therapy medicinal products (ATMPs) (Fig. 1).

APOSEC is the secretome released by cultured, stressed PBMCs. Content analysis has identified a wide spectrum of proteins, exosomes, lipids, phospholipids, and cholesterols as well as antimicrobial peptides. The topical application of APOSEC augments wound healing in an *in vivo* rodent model as well as in a porcine burn wound model^9,10^. Mode- of-action investigations revealed that APOSEC causes platelet inhibition and vasodilation, augments cytoprotective and anti-apoptotic gene product in primary cultured human cells, induces migration of fibroblasts, keratinocytes, and proliferation of endothelial cells, suppresses proliferation of highly purified CD4+ T cells, exerts antibacterial activity, and causes aortic and spinal cord endothelial cell sprouting *in vitro*^11^. APOSEC also abrogates hypoxia-induced inflammation and cell damage in non-clinical models of myocardial infarction, stroke, spinal cord injury, and myocarditis^12–14^.

Regulatory authorities classify APOSEC as a biological medicinal product (Directive 2001/83/EC), and non-clinical safety assessment thus falls under the scope of ICH S6. During development, autologous APOSEC was tested in a randomised phase I safety and tolerability study, MARSYAS I (ClinicalTrials.gov Identifier: NCT02284360). This autologous APOSEC was produced under Good Manufacturing Practice (GMP) conditions and applied topically in healthy volunteers in combination with NuGel, as described in Simader *et al*.^15^. No therapy-related serious adverse events occurred in any participants, and both low-dose (12.5 U/mL) and high-dose (25 U/mL) treatments were well tolerated. However, the production of an autologous blood product is complex and costly in terms of labour and time. Therefore, APOSEC was developed as an allogeneic product for off-the-shelf use and more favourable cost of manufacture, and to include viral clearance^16^. Allogeneic APOSEC is produced in the same way as the autologous variant under GMP conditions in the Red Cross Blood Transfusion Service of the Red Cross of Upper Austria, Linz, Austria. Up to 120 donors are pooled, which not only is important for upscaling the manufacturing product but also minimises batch-to-batch variability.

**Figure 2:**
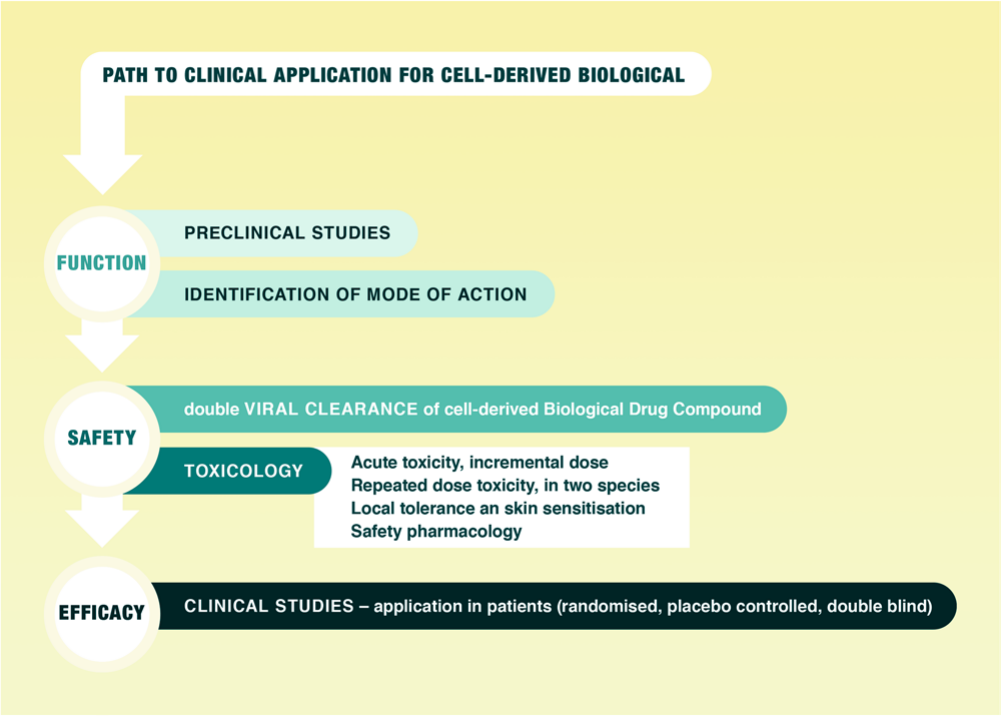
Path to clinical application for a cell-derived biological. Mandatory regulatory elements to achieve market authorisation.

APOSEC is intended for topical use on full-thickness diabetic foot ulcer (DFU) in the clinical first-in-human/phase II study MARSYAS II. The non-clinical development strategy to support the proposed phase II clinical study is primarily based on the regulatory requirements of ICH S6 (R1) ‘Preclinical safety evaluation of biotechnology-derived pharmaceuticals’ (EMA/CHMP/ICH/731268/1998) and – where relevant – on ICH guideline M3 (R2) on ‘Non- clinical safety studies for the conduct of human clinical trials and marketing authorisation for pharmaceuticals’ (EMA/CMPM/ICH/286/1995) (Fig. 2). Non-clinical studies conducted consist a range of pharmacological and toxicology studies, in which APOSEC was investigated *in vitro* and *in vivo*. APOSEC was administered topically with hydrogel as carrier in non-clinical wound-healing models^9,10^, mimicking the intended route of administration in humans.

For safety and toxicity testing, the intravenous (i.v.) and subcutaneous (s,c,) routes of administration were selected for assessing systemic toxicity. APOSEC is intended for topical use, however, to mimic clinical use for diabetic foot ulcers, the s.c. route is considered more relevant, and the selected parenteral route allows for the elucidation of potential target organs of toxicity.

Mouse and rat were selected as relevant rodent species, and the minipig was selected as a relevant non-rodent species. According to ICH S6 acute and repeated dose toxicity and safety pharmacology studies are relevant. Acute toxicity was performed in mice (iv), and repeated-dose toxicity in mice (i.v.) and minipigs (s.c.). For local tolerance testing, the local lymph node assay (LLNA) in mice was chosen. A safety pharmacological study (Irwin test) was conducted in rats.

Immunogenicity assessments in nonclinical animal studies are not relevant in terms of predicting potential immunogenicity of human or humanized proteins in humans. The administration of the human clinical product into an immune competent animal is a xenogenic situation and any generated immunogenic response does not reflect the clinical setting. Moreover, the systemic exposure to the product is expected to be low after topical application in patients and therefore the immunogenic potential risk is assumed to be low. Nevertheless, the potential for immunotoxicity was assessed as part of the pivotal repeated dose toxicity studies in mice and minipigs, investigating any hematological changes, alterations in immune system organ weights and histology, changes in serum globulins, a potential increased incidence of infections, clinical chemistry, gross pathology, organ weights and histopathology.

This report describes the toxicological preclinical studies necessary for a biological such as APOSEC, a secretome of stressed PBMCs, to be approved for a clinical Phase II study.

## Methods

APOSEC was produced at the Red Cross Blood Transfusion Service of the Red Cross of Upper Austria, Linz, Austria. Toxicological experiments were conducted at the LPT Laboratory of Pharmacology and Toxicology, GmbH & Co. KG, Hamburg, Germany.

All animal studies reported in this publication were approved by “Behörde für Gesundheit und Verbraucherschutz, Amt für Verbraucherschutz, Lebensmittelsicherheit und Veterinärwesen”, Hamburg, Germany (reference numbers V1/591-00.33, V1305-591-00.33, V1307-591-00.33, and V1305-591-00.33) and/or by “Ministerium für Energiewende, Landwirtschaft, Umwelt und ländliche Räume”, Kiel, Germany (reference number V244-27284/2015-722.04) and performed based on Guidelines from the Guide on the Care and Use of Laboratory Animals.

### Preparation of APOSEC according to GMP regulations

Blood donor selection and blood donations are in compliance with European and national regulations and standards regarding acceptance criteria, procedures, and documentation. Informed consent was obtained from all donors. For the manufacture of APOSEC, blood donations of selected donors who match important quality criteria (e.g. <45 years old, CRP <2 mg/dl, erythrocytes between certain limits, etc.) are used. PBMCs are extracted from these donations (buffy coat, density centrifugation using lymphocyte separation medium), washed twice with Dulbecco’s phosphate-buffered saline, and re- suspended with a culture medium (CellGenix GMP DC Medium). The PBMCs are driven into apoptosis by 60 Gy (2 × 30 Gy) irradiation, followed by cell culture for 24 h ± 2 h at a standardised cell density of 25 × 10^6^ cells per millilitre. After centrifugation, the PBMCs are removed, and the supernatant, including the secretome of the PBMCs, is sterile filtered. Several in process controls are implemented to ensure quality, to standardize the manufacturing process and therefore to minimize batch to batch variations. The donations, which are processed individually up to this step, are pooled and subjected to a first viral reduction step (methylene blue/light treatment). Afterwards, the product is stored at −80 °C before the process is resumed, which entails lyophilisation as well as a second, orthogonal viral reduction step (irradiation with 25–35 kGy). The latter also serves as terminal sterilisation. Subsequently, the vials are stored again at −80 °C. APOSEC is the supernatant of 25 × 10^6^ irradiated PBMCs, which are removed during the processing. The concentration of the final product is 25 U/mL, based on the cell number. However, APOSEC is a cell-free product. Lower concentrations are obtained by diluting the 25 U/mL, and higher concentrations are obtained by dissolving the lyophilisate in a smaller amount of physiological saline.

### Acute toxicity study of APOSEC by single i.v. administration to CD rats

The aim of this study was to establish the toxicity of APOSEC following single i.v. administration to CD rats. The study was performed in compliance with Note for Guidance for single-dose toxicity (3BS1a) from August 1987, published April 2001 (Directive 75/318/EEC as amended), Directive 2003/63/EC amending Directive 2001/83/EC of the European Parliament and of the Council on the Community, Safety Guideline ICH M3 (Non- Clinical Safety Studies for the Conduct of Human Clinical Trials for Pharmaceuticals, CPMP/ICH286/95 mod. released November 2000), and the U.S. Food and Drug Administration guidance on single-dose acute toxicity testing for pharmaceuticals (FDA Guidance, August 1996). In addition, the ‘Good Laboratory Practice’ regulations were considered.

Three dose-level groups (50 U, 250 U, and 500 U/kg b.w.) and one vehicle control group (0.9% NaCl; B. Braun, Melsungen, Germany) were tested. Groups of 10 animals (5 male and 5 female rats, Crl:CD(SD), Charles River Laboratories, Sulzfeld, Germany) each were examined. The animals were randomised before use. APOSEC and the vehicle were administered i.v., with 2 mL/kg b.w. injected within 2–3 minutes. Following administration, observations were made and recorded systematically, with individual records being maintained for each animal. Observations were performed before and immediately after administration and at 5, 15, 30, and 60 min and 3, 6, and 24 hours after administration. All animals were observed for a period of 14 days. During the 2-week follow-up period, observations were performed at least once a day. Mortality and changes in skin and coat, eyes, mucous membranes, respiratory and circulatory function, autonomic and central nervous system and somatomotor activity, and behavioural patterns were recorded. Attention was also paid to possible tremors, convulsions, salivation, diarrhoea, lethargy, sleep, and coma. Individual body weights were recorded before administration of the test item and thereafter in weekly intervals up to the end of the study. Changes in body weight were calculated and recorded. At the end of the experiments, all animals were sacrificed, dissected, and inspected macroscopically. All gross pathological changes were recorded. No microscopic examination was carried out because no pathological findings were noted at necropsy. No statistical analysis was performed.

### Skin sensitisation: LLNA — BrdU-ELISA of APOSEC in CBA/JN mice

The aim of this study was to determine the skin-sensitising properties of the test item with the LLNA BrdU-ELISA in mice. The study was carried out according to the OECD Guideline for the Testing of Chemicals No. 442B, Skin Sensitisation: Local Lymph Node Assay: BrdU- ELISA, adopted July 22, 2010, and according to Commission Regulation (EU) No. 640/2012, method B.51 of July 6, 2012. The ‘Good Laboratory Practice’ regulations were also considered.

APOSEC was reconstituted and further diluted with 0.9% aqueous NaCl solution. Three test item doses with secretome supernatant at 25 U/mL, 125 U/mL, or 250 U/mL were tested in five female CBA/JN mice (Janvier Labs, Saint Berthevin Cedex, France) per group and compared to a vehicle control group (NaCl alone). As a positive control group, animals were treated with a 25% solution (v/v) of α-hexyl cinnamic aldehyde (Sigma-Aldrich Chemie GmbH, Traufkirchen, Germany) in acetone (Sigma-Aldrich)/olive oil (Caesar & Loretz GmbH, Hilden, Germany) (4:1 v/v). Additionally, one group received only the vehicle of the positive control. The test item solutions were applied on the dorsal surface of ears of experimental animals (25 µL/ear) for three consecutive days (days 1, 2, and 3). There was no APOSEC treatment on days 4, 5, and 6. On day 5, a total of 0.5 mL (5 mg/mouse) of BrdU (10 mg/mL) (Sigma-Aldrich) solution was injected inter-peritoneal. On day 6, animals were humanely killed. The draining auricular lymph nodes from each mouse ear were excised and processed separately in phosphate buffered saline (GIBCO Invitrogen GmbH, Karlsruhe, Germany) for each animal. From each mouse, a single-cell suspension of lymph node cells (LNCs) excised bilaterally was prepared by gentle mechanical disaggregation through 200-µm mesh stainless steel gauze followed by passage through a 74-µm nylon mesh. The target volume of the LNC suspension was adjusted to a determined optimized volume (approximately 15 mL). The optimized volume was based on achieving a mean absorbance of the LNC group within 0.1–0.2 (measured with a spectrophotometer at λ = 570 nm). The cell proliferation in the local lymph nodes was determined by measuring the BrdU content with BrdU-ELISA using a commercial kit (Roche Applied Science, Mannheim, Germany). Values obtained were used to calculate stimulation indices.

Animals were observed once daily for any signs of local systemic irritation at the application site or signs of systemic toxicity. Observations were recorded for each individual animal. Cage-side observations included skin/fur, eyes, mucous membranes, respiratory and circulatory systems, somatomotor activity, and behaviour patterns. The onset, intensity, and duration of any signs observed were recorded. The weight of each mouse was recorded at the time of allocation of animals to groups (test day 1) and at the time of necropsy (test day 6).

### Neuropharmacological screening in rats according to Irwin, following i.v. administration of APOSEC

The aim of this experiment was to examine the effect of APOSEC on neuropharmacological parameters in male and female rats using a neuropharmacological screening test following i.v. administration. The study was performed in compliance with ICH S7A (Safety Pharmacology Studies for Human Pharmaceuticals, July 2001), ICH M3(R2) (Non-Clinical Safety Studies for the Conduct of Human Clinical Trials for Pharmaceuticals, December 2009), EEC Council Directive 2001/83/EC, and subsequent amendments. The ‘Good Laboratory Practice’ regulations were considered.

Five groups of six male and six female rats (Crl:CD (SD), Charles River Laboratories, Sulzfeld, Germany) each were examined. One negative control group (0.9% aqueous NaCl), three test item–treated groups administered concentrations of 50 U, 250 U, or 500 U/kg b.w., and one positive control group of 8 mg diazepam/kg b.w. (Sigma-Aldrich) were tested. The vehicle control and test item–treated groups were administered i.v. into a tail vein, and the positive control was administered orally. The administration volume was 2 mL/kg b.w. The possible influence of APOSEC was examined on 40 neuropharmacological parameters (behavioural reactions, motor activity, central nervous system, posture, motor coordination, muscle tone, reflexes) pre-dose and at 1, 4, 7, 24, 48, and 72 hours after administration.

### 4-week toxicity study of APOSEC by i.v. slow bolus administration three times weekly to mice

The aim of the study was to obtain information about the toxicity and local tolerance of APOSEC after repeated i.v. slow bolus administration (three times weekly) to C57BL/6N mice for 4 weeks and to assess the reversibility of any effect at the end of a 2-week recovery period.

#### Animals

C57BL/6N mice were supplied by Charles River Laboratories (Germany GmbH). The body weight range did not exceed 10% of the mean weight for each sex at the time of selection. An initial health check was performed upon delivery of the animals. Only animals free of signs of illness were selected for the study. The animals were allocated to the four study groups based on body weight by means of a computerized randomisation program. Animals with a body weight at the extremes of the weight distribution were excluded and replaced by healthy spare animals. The animals in the main study (40 males and 40 females) were randomised into 10 animals/sex/group. Recovery animals were additionally assigned to groups 1 (control group) and 4 (high-dose group), respectively, with 5 male and 5 female animals each. The mice were treated with 50, 250, or 500 Units APOSEC/kg b.w. The control animals received the vehicle (sterile 0.9% NaCl solution).

#### Diet and housing

The commercial diet ssniff-R/M-H V1534 (ssniff Spezialdiäten GmbH, 59494 Soest, Germany) was used for feed and offered *ad libitum*. Food residue was removed and weighed weekly. Drinking water was offered *ad libitum*. The animals were kept singly in Makrolon cages (type II) at room temperature (22 °C ± 3 °C) and a relative humidity of 55% ± 10%. The rooms were lit and darkened for periods of 12 hours each. The air change rate was 15–20 exchanges/hour.

#### Preparation of APOSEC

The APOSEC was reconstituted with sterile 0.9% aqueous NaCl solution to the appropriate concentration as described in Table 1. For each dose level, separate bottles/vials were reconstituted. The administration solutions were freshly prepared on each administration day before initiation of the administration.

**Table 1:** Concentrations of APOSEC in the mouse i.v. study.

Dose level [Units/kg]	Content per test item vial [U]	Vial filled to a final volume of [mL]	Achieved concentration of the administration solution [U/mL]	Treatment group
50	500	20	25	2
250	500	4	125	3
500	500	2	250	4

#### Administration

Three times weekly for 4 weeks (in total 13 administrations), 2 mL/kg b.w. were injected by an i.v. slow bolus administration into the tail vein. Sterile 0.9% NaCl solution served as vehicle. The test item formulations were administered i.v. at a constant relative administration volume. The control animals received the vehicle at the same administration volume in the same way as the high-dose group. The amount of test item was adjusted to each animal’s current body weight on each administration day.

#### Group size/Dose levels

The animals were allocated to the four test groups based on body weight by means of a computer-generated randomisation program (Table 2).

**Table 2:** Group size and dose level of APOSEC in the mouse i.v. study.

Group	APOSEC dose, i.v. [U/kg b.w.]		Number of animals and sex MS (+RP)
1	0	(0.9% NaCl solution)	10 (+5) m 10 (+5) f
2	50	(low dose)	10 m 10 f
3	250	(intermediate dose)	10 m 10 f
4	500	(high dose)	10 (+5) m 10 (+5) f

B.w., body weight; i.v., intravenous; MS, main study; RP, recovery period; m, male; f, female.

#### Observations

Clinical signs: The animals were observed individually before and after dosing at each time of dosing for any signs of behavioural changes, reaction to treatment, or illness. In addition, the animals were checked regularly throughout the follow-up period. Cage-side observations included skin/fur, eyes, mucous membranes, respiratory and circulatory systems, somatomotor activity, and behaviour patterns. The onset, intensity, and duration of any signs observed were recorded. Dated and signed records of appearance, change, and disappearance of signs were maintained on clinical history sheets for individual animals. Special attention was paid to the local tolerance of the test item at the injection sites. Body weight and food and drinking water consumption were monitored.

Laboratory examinations: Blood samples were taken from the retrobulbar venous plexus under isoflurane anaesthesia from animals fasted overnight. The blood samples collected were divided into tubes as follows: EDTA anticoagulant (whole blood) for haematological investigations; citrate anticoagulant (plasma) for coagulation tests; and Li-heparin anticoagulant (plasma) for clinical chemical tests.

Urinalysis: Urine samples were collected from animals fasted overnight on day 29 from all animals in the main study and on test day 43 from all recovery animals. Because of the low excretion volume of urine per animal, mice were placed in Urimax funnel cages in groups of 5 animals per sex. After the mice had received 30 mL tap water/kg b.w. by gavage, urine was collected for 6 hours.

Ophthalmological and auditory examinations: Examinations were performed before the first administration and on test day 29 (at the end of the treatment period) for all animals as well as on day 43 (at the end of the recovery period) for all recovery animals. The eyes were examined with a Heine ophthalmoscope. After examination of the pupillary reflex, mydriasis was produced by instillation of Stulln eye drops onto the cornea. The following ocular structures were then examined: adnexa oculi (i.e., lids, lacrimal apparatus), conjunctiva, cornea, anterior chamber, lens, vitreous body, and fundus (retina, optic disc). Auditory acuity was checked with a simple noise test.

#### Pathology and histopathology

On test day 30 (approximately 24 hours after the last administration), the main study animals were dissected following a randomisation scheme. Necropsy of all animals assigned for the recovery period was carried out on test day 44.

Immediately after blood withdrawal for laboratory examinations, the animals were sacrificed under inhalation anaesthesia (isoflurane), weighed, dissected, and inspected macroscopically under the direction of a pathologist. Additionally, organs were examined histopathologically after preparation of haematoxylin–eosin-stained paraffin sections, and frozen sections of the heart, liver, and one kidney were prepared, stained with Oil Red-O, and examined histopathologically. During dissection, fresh bone marrow was obtained from the os femoris (3 air-dried smears/animal) of 5 main study animals per sex and group and of all recovery animals, and stained according to Pappenheim. The myeloid:erythroid ratio was determined by cell differentiation (counting of 200 nuclei-containing cells).

#### Statistics

Test item groups 2 to 4 were compared to control group 1. The following statistical methods were used: multiple t-tests based on Dunnett’s for body weight, food consumption, haematology, coagulation, clinical chemistry, and relative and absolute organ weights (p ≤ 0.05 and p ≤ 0.01); chi^2^ test for bone marrow observations (p ≤ 0.01); and the Fisher’s exact test for histopathology data (p ≤ 0.05). Homogeneity of variances and normality of distribution were tested using the Bartlett’s and Shapiro–Wilks tests, respectively. In case of heterogeneity and/or non-normality of distribution, stepwise transformation of the values into logarithmic or rank values was performed prior to analysis of variance (ANOVA). If the ANOVA indicated a significant effect (p ≤ 0.05), intergroup comparisons with the control group were made by the Dunnett’s test (p ≤ 0.01 and p ≤ 0.05). These statistical procedures were used for all data.

### 4-week toxicity study of APOSEC in Aachener minipigs with s.c. administration three times weekly

The aim of this study was to obtain information on the toxicity of APOSEC in Aachener minipigs administered APOSEC by s.c. bolus injection three times weekly for 4 weeks and to assess the reversibility of any effect after a 2-week recovery period. The minipig was selected as a suitable animal species because of the similarities of its skin to that of humans. Porcine and human skin have many structural features in common: sparse haircoat, absence of pigmentation, structural appearance of the surface, general morphology, comparable epidermal thickness and epidermal cell turnover time, orientation and distribution of vessels, and a similar immunological reactivity. Minipig skin offers the best prediction of the transdermal penetration and absorption in human. In addition, the minipig skin is less sensitive to irritants than rabbit skin and thus offers a better prediction for adverse effects in humans than do studies in rabbits or other species. The abundant surface area of the minipig also allows multiple-site and long-term testing, which is usually not possible in rabbit and rodent species.

Animals and housing: Naïve Aachener minipigs (Gerd Heinrichs, Heinsberg, Germany) were used in this experiment. For the main study, 24 animals (12 males, 12.6–18.0 kg; 12 females, 11.8–16.7 kg) were randomised to 3 animals/sex/group. Eight recovery animals (4 males and 4 females) were randomised to 2 animals/sex for groups 1 (control group) and 4 (high-dose group).

The pigs were kept singly in indoor pens. The rooms were lit and darkened for periods of 12 hours each. Commercial ssniff MPig-H Ered V4173 (ssniff Spezialdiäten GmbH, 59494 Soest, Germany) served as food. The animals were fed with a suitable amount according to their age and body weight (as recommended by the breeder G. Heinrichs, Germany) once daily. Tap water of drinking water quality was offered *ad libitum*.

#### Preparation of the administration solutions

The lyophilised test item was reconstituted with sterile 0.9% aqueous NaCl solution to the appropriate concentration. Each vial containing the lyophilisate was reconstituted with 20 mL 0.9% NaCl to yield a concentration of 25 U/mL. The administration solutions were freshly prepared on each administration day. Details are given in Table 3.

**Table 3:** Concentration of APOSEC in the minipig s.c. study.

Dose level [U/kg b.w.]	Content per test item vial [U]	Concentration [U/mL]	Administration volume [mL/animal]	Administration volume [mL/kg b.w.]
3.3	500	25	1.98	0.132
10.5	500	25	6.30	0.420
33.3	500	25	19.80	1.332

D1: 3.3 U/kg; max 15 kg = 49.5 Mio/animal = 1.98 mL.

D2: 10.5 U/kg; max 15 kg = 157.5 Mio/animal = 6.30 mL.

D3: 33.3 U/kg; max 15 kg = 495.0 Mio/animal = 19.80 mL.

#### Administration

APOSEC was administered by s.c. bolus injection under the skin in the inguinal region of the left and right sides (alternating, starting with the left side) three times weekly for 4 weeks (a total of 13 administrations). The control animals received the vehicle at the same administration volume in the same way as the high-dose group. The amount of the test item was adjusted to the animal’s body weight on each administration day (Table 4). The animals were allocated to the four test groups by means of a computer-generated randomisation program.

**Table 4:** Group size and dose level of APOSEC in the minipig s.c. study.

Group	APOSEC dose [U/kg b.w., s.c.]	Number of animals and sex MS (+RP)
1	0 (0.9% NaCl solution)	3 (+2) m 3 (+2) f
2	3.3 (low dose)	3 m 3 f
3	10.5 (intermediate dose)	3 m 3 f
4	33.3 (high dose)	3 (+2) m 3 (+2) f

B.w., body weight; s.c., subcutaneous; MS, main study; RP, recovery period; m, male; f, female.

#### Observations

Local tolerance: After each dosing, the injection sites were checked for obvious signs of local intolerance. When such signs were noted, the sites were monitored daily during the treatment period until the changes had subsided. A final check was made at the end of the treatment-free recovery period.

Clinical signs: The animals were observed individually before and after dosing at each time of dosing for any signs of behavioural changes, reaction to treatment, or illness. Pen-side observations included skin/fur, eyes, mucous membranes, respiratory and circulatory systems, somatomotor activity, and behaviour patterns. The onset, intensity, and duration of any signs observed were recorded. Body weight and food and drinking water consumption were monitored.

Non-invasive telemetry employing the EMKA system: The animals were habituated during the acclimatisation period to the telemetry procedures and trained to wear the necessary telemetry jackets, electrodes, and cuffs, as well as to the general handling procedures and influences (e.g., the sound of the blood pressure pump) during telemetric data collection. In a step-wise approach, the animals learned to accept and wear the telemetry jacket carrying the transmitter device and blood pressure pumps (daily prolongation of wearing time). In this context, the animals were also trained to tolerate shaving and cleansing of the chest and legs for electrode patches and the blood pressure cuff. The animals were trained for at least one week. If necessary, training was prolonged for individual animals. Parameters were monitored telemetrically with the EMKA Technologies System continuously starting approximately 24 hours prior to treatment (baseline) on 1 occasion (test day −1) and for 24 hours following injection on days 1 and 29, i.e., for a total of 48 hours at study start and for approximately 24 hours after the final administration. Electrocardiography (ECG), skin temperature, respiratory, and physical activity data were recorded continuously. Blood pressure data were transmitted in four consecutive 2-minute intervals followed by a 12-minute pause. As blood sampling can cause a disturbance in the ECG recordings, the ECG was evaluated at a time sufficiently before or after the scheduled blood sampling time.

Laboratory examinations: The blood samples for haematological (including coagulation) and biochemical examinations were taken from the vena jugularis from animals fasted overnight at baseline (pre-dose), on day 30 (i.e., at the end of the treatment period, 32 animals in the study) and on day 43 (i.e., at the end of the 2-week recovery period, 8 recovery animals). For haematological examinations, EDTA anticoagulant (whole blood) was used; citrate anticoagulant (plasma) was used for coagulation tests; and serum (no anticoagulant) was collected for clinical biochemistry tests.

Urinalysis: Urine samples were collected in the morning of the respective test day in a metabolism cage for 3 hours prior to the administration after the animals received 50 mL tap water/kg b.w. by gavage. Samples were drawn at baseline (pre-dose) and on day 28 (at the end of the treatment period, all animals) and day 42 (at the end of the 2-week recovery period, 8 recovery animals).

Ophthalmological and auditory examinations: The examinations were performed at day 1 (pre-dose, before first administration) and day 30 at the end of the treatment period. The eyes were examined with a Heine ophthalmoscope. Prior to the examinations listed below, the pupillary reflex was examined. Thereafter, mydriasis was produced by instillation of Stulln eye drops onto the cornea. Afterwards, ocular structures were examined: adnexa oculi (i.e., lids, lacrimal apparatus), conjunctiva, cornea, anterior chamber, lens, vitreous body, and fundus (retina, optic disk). Auditory acuity was checked with a simple noise test.

#### Pathology and histopathology

Necropsy: On test day 30 (approximately 24 hours after the last administration), all main study animals were dissected following a randomisation scheme. Necropsy of all animals assigned for the 2- week recovery period was carried out on test day 44. The animals (fasted overnight) were sacrificed (150 mg pentobarbital/kg b.w., i.v.) and exsanguinated by carotid dissection, weighed, dissected, and inspected macroscopically under the direction of a pathologist.

Histopathology: The organs were examined histopathologically after preparation of paraffin sections and haematoxylin–eosin staining. Parathyroids were examined microscopically if in the plane of section. In addition, frozen sections of the heart, liver, and of one kidney from all animals were made, stained with Oil Red-O, and examined microscopically. Furthermore, a histopathological examination was performed on a Ki67-immunostained section of the injection site of each animal of the control and high-dose groups (n = 10 animals per group).

Bone marrow evaluation: During dissection, fresh bone marrow was obtained from a suitable rib, e.g., the 7th rib (3 air- dried smears/animal) of all animals (main study and recovery animals) and stained according to Pappenheim.

#### Statistics

The test item groups 2 to 4 were compared to the control group (group 1). The following statistical methods were used: multiple t-tests based on Dunnett for body weight, food consumption, telemetry data, haematology, coagulation, biochemistry, urinalysis, and relative and absolute organ weights (p ≤ 0.01 and p ≤ 0.05), and Fisher’s exact test for histology data (p ≤ 0.05). The following settings were used for the statistical evaluation of the parametrical values captured by Provantis: homogeneity of variances and normality of distribution were tested using the Bartlett’s and Shapiro–Wilks test for all data. In case of heterogeneity and/or non-normality of distribution, stepwise transformation of the values into logarithmic or rank values was performed prior to ANOVA. If the ANOVA indicated a significant effect (p ≤ 0.05), intergroup comparisons with the control group were made by the Dunnett’s test. These statistical procedures were used for all data.

## Results

### Acute toxicity study of APOSEC by single i.v. administration to CD rats

Under the present test conditions, single i.v. administrations of different doses of APOSEC ranging from 50 U/kg b.w. to 500 U//kg b.w. did not reveal any signs of toxicity. No animal died prematurely. All animals gained the expected body weight throughout the whole study period. There were no macroscopic findings during necropsy. The no-observed-effect level (NOEL) was defined at or above the highest tested dose of 500 U/kg b.w. administered by i.v. administration to rats.

### Skin sensitisation: LLNA — BrdU-ELISA of APOSEC in CBA/JN mice

The stimulation indices of the test item–treated groups calculated for the BrdU labelling index did not exceed the threshold value of 1.6. Hence, the test item is classified as not skin sensitising (Table 5).

**Table 5:** Results of LLNA of APOSEC in mice.

Parameter	Group 1 Negative control	Group 2 25 U/kg b.w.	Group 3 125 U/kg b.w.	Group 4 250 U/kg b.w.	Group 5 Positive control	Group 6 Vehicle of positive control
BrdU labelling	1.000	1.216	1.175	1.279*	1.831*	1.419*
Ear weight	1.000	0.965	0.913	0.913	1.070*	0.890
Difference in ear thickness (day 3)	1.000	0.976	1.010	1.005*	1.208*	1.020
Difference in ear thickness (day 6)	1.000	0.995	1.010	1.005*	1.130*	1.058*

*Significant increase compared to control at p ≤ 0.01.

There were no deaths, and no systemic clinical signs or effects on body weights were observed during the study. The ear weight (punch biopsies) and the difference in ear thickness on test day 3 and test day 6 compared to the vehicle control were not or only slightly increased, i.e., no skin-irritating properties were noted. Treatment with the positive control item caused the expected increases in the BrdU labelling index. Therefore, the study can be regarded as valid. No signs of systemic intolerance were recorded. Treatment did not affect animal body weight. The present test conditions revealed no skin-sensitising properties of the test item APOSEC in the LLNA.

### Neuropharmacological screening in rats according to Irwin following i.v. administration of APOSEC

The possible influence of APOSEC at dosages of 50, 250, and 500 U/kg b.w. was examined on 40 neuropharmacological parameters pre-dose and at 1, 4, 7, 24, 48, and 72 hours after administration. Compared to the vehicle control (group 1), 17 neuropharmacological parameters were influenced in a dose-related way and noted at 1 hour after administration at doses of 250 and 500 U/kg b.w. (groups 3 and 4, Table 6). All values fully recovered within the next 3 hours. No findings were observed in the animals treated with the low dose of 50 U/kg b.w. (group 2) or the negative control substance (vehicle 0.9% NaCl solution). By comparison, the positive control diazepam (8 mg/kg b.w., peroral) influenced 19 of 40 parameters. The temporary impairments in these parameters observed at 1 hour after administration were an immediate consequence of the high i.v. doses, which have a particular impact on the parameters of vitality. In all other animal studies performed in mice, rats, and minipigs, these effects were not observed at this intensity.

**Table 6:** Changes in neuropharmacological parameters compared to group 1 (control) in treatment groups 2, 3 and 4 one hour after administration. Data are given in deviation to normal value.

		Group 2	Group 3	Group 4
Awareness	Alertness	0	−1	−1
	Visual placing	0	−4	−4
	Passivity	0	+2	+2
	Stereotypy	0	0	0
Mood	Grooming	0	−4	−4
	Vocalisation	0	0	0
	Restlessness	0	0	0
	Aggression	0	0	0
Motor activity	Reactivity	0	0	−4
	Spontaneous acitvity	0	−1	−2
	Touch response	0	−4	−4
	Pain response	0	−2	−2
CNS excitation	Startle response	0	0	−2
	STRAUB tail	0	0	0
	Tremor	0	0	0
	Twitching	0	0	0
	Convulsion	0	0	0
Posture	Body posture	0	−4	−4
	Limb position	0	−4	−4
	Staggering gait	0	+2	+2
	Abnormal gait	0	0	0
	Righting reflex	0	+1	+3
Muscle tone	Limb tone	0	0	0
	Grip strength	0	0	0
	Body sag	0	0	0
	Body tone	0	−1	−2
	Abdominal tone	0	−1	−2
Reflexes	Pinna	0	0	−4
	Corneal	0	0	0
	Ipsilateral Flexor Reflex	0	0	0
Autonomic function	Writhing	0	0	0
	Pupil size	0	0	0
	Palpebral opening	0	−2	−2
	Exophthalmus	0	0	0
	Urination	0	0	0
	Salivation	0	0	0
	Piloerection	0	0	0
	Hypothermia	0	0	0
	Skin colour	0	0	0
	Respiration rate	0	0	0

### 4-week toxicity study of APOSEC by i.v. slow bolus administration three times weekly to mice

**Figure 3:**
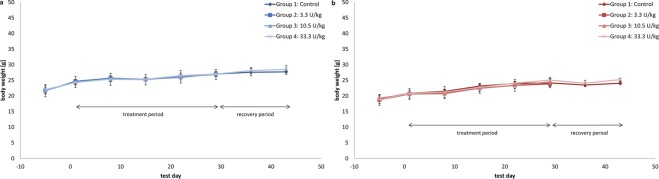
Body weight and body weight gain of male (**a**) and female (**b**) mice. Data are given in mean values per group.

APOSEC was applied three times weekly for 2 weeks to mice at dosages of 50, 250, and 500 U/kg b.w. via i.v. slow bolus injection into the tail vein. No signs of local intolerance reactions were observed in any dose group during the study. Neither the macroscopic inspection at necropsy nor the histopathological examination (restricted to the control and high-dose groups) revealed any test item–related changes at the injection sites of the animals. None of the animals died or had to be sacrificed prematurely. No signs of systemic toxicity or behavioural effects were noted during the study. No test item–related influence on body weight was noted (Fig. 3). Neither haematological, nor clinical chemistry (Table 7) parameters showed test item related alterations compared to the control group. Histopathological examinations of the main study animals, previously high-dose recovery animals, and control animals revealed no local intolerance or systemic morphological lesions that were considered to be test-item related. Under the test conditions of this study, the NOEL was above 500 U allogenic APOSEC/kg b.w. three times weekly for 4 weeks (13 applications) by i.v. slow bolus injection.

**Table 7:** Haematological and clinical chemistry parameters with significant differences compared to the control animals. Differences are not considered to be test item-related.

Parameter	Group/Sex	mean value SD	Test day	Statistical significance	Reason
Reticulocytes (%)	4 f	4.15 0.68	25	p ≤ 0.01	A
Platelets (x10^3^/µl)	4 m	1085.0 55.5	25	p ≤ 0.01	A
Eosinophilic granulocytes (absolute; x10^3^/µl)	4 f	0.266 0.064	39	p ≤ 0.05	A
Thrombin time (s)	4 m	13.40 0.37	44	p ≤ 0.05	A
ALAT (U/L)	4 m	43.8 10.0	44	p ≤ 0.05	A
ASAT (U/L)	4 m	124.6 27.6	44	p ≤ 0.05	A

m: male.

f: female.

A: the slight alteration in comparison to control animals is without any biological relevance.

ALAT: alanine aminotransferase.

ASAT: aspartate aminotransferase.

### 4-week toxicity study of APOSEC in Aachener minipigs with s.c. administration three times weekly

#### Local tolerance

None of the animals treated with 3.3, 10.5, or 33.3 U/kg b.w. three times weekly by s.c. injection revealed any test item–related signs of local intolerance during the 4-week treatment period or the 2-week treatment-free recovery period (Tables 8 and 9).

**Table 8:** Assessment of local intolerance reactions according to DRAIZE.

Macroscopic changes at the injection sites of the main study animals at terminal sacrifice on test day 30 (animals affected/animals examined)								
Finding	Group 1 Control		Group 2 3.3 U/kg		Group 3 10.5 U/kg		Group 4 33.3 U/kg	
	m	f	m	f	m	f	m	f
Haemorrhage, s.c.	0/3	0/3	0/3	2/3	1/3	1/3	1/3	1/3
Thickened	0/3	0/3	0/3	0/3	0/3	1/3	0/3	0/3

m: male.

f: female.

s.c.: ubcutaneous.

**Table 9:** Local intolerance in form of reddenings, swellings and/or thickenings of the subcutaneous injection sites.

Local intolerance reactions during the treatment period (animals affected/animals examined)								
Finding/Incidence	Group 1 Control		Group 2 3.3 U/kg		Group 3 10.5 U/kg		Group 4 33.3 U/kg	
	m	f	m	f	m	f	m	f
Discoloured, reddened or thickened	0/5	0/5	0/3	1/3	1/3	2/3	0/5	1/5
noted on up to ‘x’ test days	—	—	—	1	6	2	—	1

m: male.

f: female.

The histopathological examination of the tissue slides of the injection sites did not reveal microscopic changes related to treatment with the test item. Hence, all changes noted at the injection sites of the male and female animals during the in-life phase of the study or at necropsy were regarded as the result of the s.c. administration procedure rather than any irritating properties of the test item. Thus, the tested concentration of 25 U/mL administered to three dose groups by s.c. injection three times weekly did not result in local intolerance reactions.

#### Systemic tolerance

**Figure 4:**
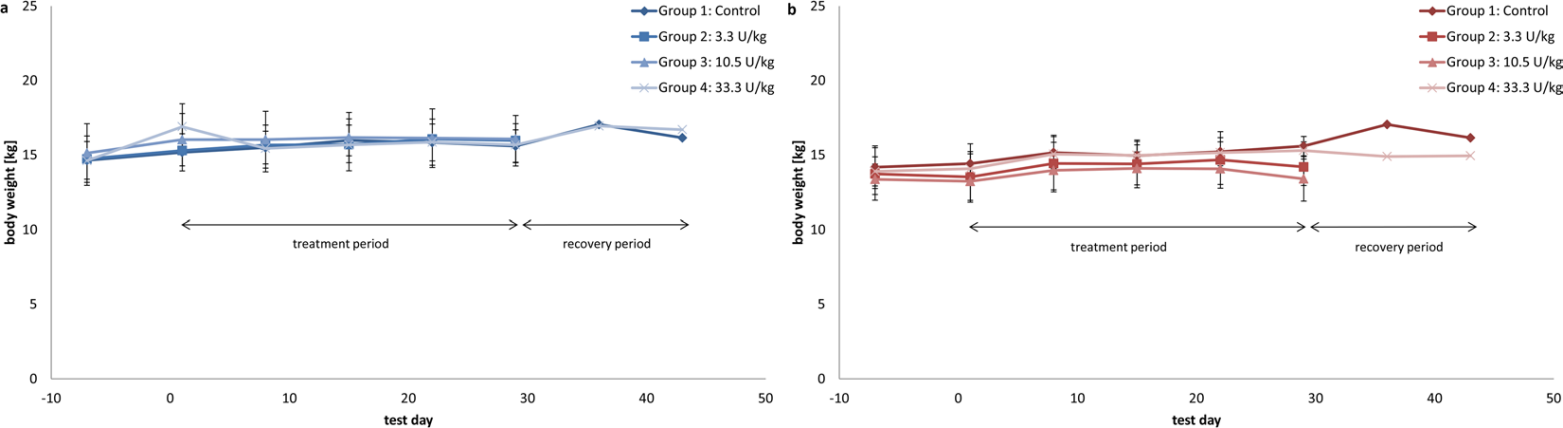
Body weight and body weight gain of male (**a**) and female (**b**) pigs. Data are given in mean values per group.

None of the animals died prematurely or revealed any test item–related signs of systemic intolerance. No test item–related changes were observed in the external appearance of the animals or for behaviour, especially none related to the central nervous system. No effects were observed on body weight (Fig. 4), food and water consumption, haematological and biochemical parameters (Table 10), urinary status (Table 11), ophthalmologic and auditory functions, or relative and absolute organ weight in any animals in any dose group. None of the animals in the different dose groups were affected based on the telemetry parameters of safety pharmacology (heart rate; RR interval; the P segment; the PQ, QRS, and QT intervals; the QTc values [according to Bazett, Fridericia and van de Water], physical activity and skin temperature, peripheral arterial systolic and diastolic blood pressure, resulting mean blood pressure, respiratory rate, or tidal volume). All parameters were within normal biological variability. Furthermore, no pathological arrhythmias were observed. Haematological, biochemical, and urinary parameters were within normal biological variation, and no test item–related changes were noted. The ophthalmological examination revealed no pathological changes in the animals at the end of the 4-week treatment or at the end of the 2-week treatment-free recovery period. There was no indication of any impairment in auditory acuity.

**Table 10:** Haematological and clinical chemistry parameters with significant differences compared to the control animals. Differences are not considered to be test item-related.

Parameter	mean value SD	Group/Sex	Test day(s)	Statistical significance	Reason
Haemoglobin (mmol/L)	9.32 0.19	4 m	−5	p ≤ 0.05	C
Leucocytes (×10^3^/µL)	11.753 1.720	3 m	30	p ≤ 0.05	A
	13.946 2.708	4 m	30	p ≤ 0.05	A
Haematocrit (%)	46.0,3 2.14	2 m	−5	p ≤ 0.05	C
	45.72 0.76	4 m	−5	p ≤ 0.05	C
Eosinophilic granulocytes (×10^3^/µL	0.317 0.072	2 m	−5	p ≤ 0.05	C
	0.303 0.065	3 m	−5	p ≤ 0.05	C
	0.273 0.075	2 m	30	p ≤ 0.01	A
	0.327 0.176	3 m	30	p ≤ 0.01	A
	0.506 0.143	4 m	30	p ≤ 0.01	A
Basophilic granulocytes	0.050 0.010	3 m	30	p ≤ 0.05	A, B
Beta-Globulin	12.97 0.12	2 m	30	p ≤ 0.05	A, B
ASAT	54.0 26.9	2 m	30	p ≤ 0.01	A, B
LDH	674.0 112.7	2 m	30	p ≤ 0.01	A, B
CK	1799.7 1789.6	2 m	30	p ≤ 0.05	A, B
GLDH	3.63 2.70	3 f	−5	p ≤ 0.01	C

m: male.

f: female.

A: the slight alteration in comparison to control animals is without any biological relevance.

B: lacking dose dependence.

C: effect observed before start of treatment.

**Table 11:** Urinary parameters with significant differences compared to the control animals.

Parameter	Increase ↑ Decrease ↓	Group/Sex	Test day	Statistical significance	Reason
Specific gravity	1.0112 0.0048	4 f	−6	p ≤ 0.05	B
pH value	6.63 0.86	2 m	28	p ≤ 0.05	A
	6.72 0.53	4 m	28	p ≤ 0.01	A
	7.62 0.19	4 f	28	p ≤ 0.05	A

Differences are not considered to be test item-related.

m: male.

A: the slight alteration in comparison to control animals is without any biological relevance.

B: effect observed before start of treatment.

#### Final examinations

The macroscopic inspection at necropsy and the histopathological examination revealed no morphological changes in male and female animals that were considered to be related to the administration of APOSEC. A small number of macroscopic changes were noted for the adrenals (reduced, n = 1), stomach (discoloured fundus mucosa, n = 1), kidneys (cyst, n = 1), lungs (discoloured, indurated, n = 1), or ovaries (cystic, n = 1) of individual male or female animals from the control and test item–treated groups, either at sacrifice at the end of the treatment period or at recovery sacrifice at the end of the recovery period. Because of the low number of animals affected, these findings are regarded as incidental changes and not related to treatment with the test item.

Furthermore, the histopathological examination revealed no test item–related microscopic changes in the respective organs. All organ weights recorded for the male and female animals employed in the study were regarded as within the normal biological range of variation for minipigs. Furthermore, no test item–related histopathological changes were noted for any of the organs examined.

No morphological difference in the bone, joints, or bone marrow was observed between control and treatment groups. Histopathological examinations also included the study of cell proliferation. During examination with Ki67, no test item–related influence on cell proliferation was noted in the epidermis, corium, or granulation tissue in the subcutis. The skin around the injection sites exhibited no proliferative processes that were considered to be related to the test item. The haematoxylin–eosin-stained sections and the Ki67 reactions showed no morphological differences between male and female animals of group 1 and the high-dose animals of group 4. Based on the above results, the NOEL was defined at or above the highest tested dose of 33.3 U APOSEC/kg b.w. administered by s.c. injection three times weekly.

## Discussion

APOSEC is a cell-derived biological medicinal product intended to promote and accelerate wound granulation and thus healing of full-thickness neuropathic, chronic, diabetic ulcers. It is the secretome of apoptotic PBMCs and therefore a cell-based but cell-free product. As already shown in several studies, the secretome of (stem) cell cultures is sufficient to accelerate cutaneous wound healing^4,5^. The idea of using conditioned medium/secretome as a therapeutic agent originated from stem cell–based therapy in myocardial infarction treatment, in which the regenerative effects following administration of stem cells are mediated via paracrine signalling rather than by direct cellular interactions^1,6^.

During the past 10 years, the detailed functions of APOSEC was investigated in several animal models and human *in vitro* settings^13,14,17–20^. Interestingly, the secretome of irradiated PBMCs acts strongly anti-inflammatory and pro-angiogenic, fostering tissue regeneration^11^. In all these experiments no immunogenic reactions were detected. In contrast to most advanced therapy medicinal products (ATMP), e.g. GMP mesenchymal stem cells, APOSEC is a completely cell free product consisting of proteins, lipids and exosomes^9,19^. Obviously, cell-free products offer some advantages compared to cell-containing products such as the possibility of viral clearance or not needing to expand the cells under what are often elaborate and costly conditions (Fig. 1).

Non-clinical studies conducted so far consist of a range of pharmacological and toxicology studies, in which APOSEC was investigated *in vitro*^11^ and *in vivo*^9,10,12–14^. Moreover, a phase I study with APOSEC has been conducted successfully in a small sample of healthy male volunteers to evaluate safety and local tolerability^15^. Nevertheless, several non-clinical studies must be conducted to prove safety prior to testing in a clinical phase II study in patients suffering DFU.

Primary pharmacodynamic testing (not reported here) has been performed *in vitro* and *in vivo*. *In vitro* assays were conducted in human dermal primary cells^19^, and APOSEC was administered *in vivo* topically in wound-healing models, mimicking the intended route of administration in humans. One was a diabetic mouse model^9^, which represents an established model of impaired wound healing, and the other was in pigs^10^ as a suitable animal species for efficacy testing because of the structural, morphological, and metabolic similarities of its skin to that of humans. *In vitro* assays showed an increased migration from dermal fibroblasts and keratinocytes, and *in vivo* results indicated that APOSEC can enhance and promote wound- healing processes. All of these data clearly indicate a potential beneficial effect of APOSEC in diabetic wound healing.

APOSEC is considered a biological medicinal product with a complex mixture of active ingredients. The metabolic pathways of peptides, proteins, and lipids are generally understood. Therefore, stand-alone pharmacokinetic studies were not conducted. However, systemic exposure of some selected human cytokines (MMP-9 and IL-8) and growth factors (MIF and EGF), which are present in the secretome of PBMCs in appreciable concentrations^17^, has been investigated in preliminary studies in rats and dogs (data not shown): Serum levels of IL-8 and MMP-9 were detectable only at 5 and 30 minutes post-administration in the rat study, measured with validated ELISAs. All serum levels of EGF and MIF were below the lower limits of quantification at any time point. These studies showed that even after single and repeated administration of APOSEC to rats, systemic exposure remained low or not quantifiable, and no accumulation of these cytokines and growth factors was detected in the circulation. In a single-dose toxicity study in beagle dogs, a dose of 250 U APOSEC/kg b.w. was applied by a slow bolus i.v. injection. The result showed that IL-8 levels were quantifiable only at 30 minutes and 3 hours post-administration (with a maximum concentration value of 24.3 pg/mL). A mean serum level of 55.6 ng MMP-9/mL was measured 5 minutes post- administration. The MMP-9 serum levels measured at 30 minutes and 3 hours post- administration were below the limits of quantification. In summary, exposure of rats to the selected candidate cytokines by repeated i.v. administration at 500 U/kg and after single i.v. administration of 250 IU/kg in dogs was low, and no accumulation was observed.

Results of a neuropharmacological screening test (Irwin) showed that a single i.v. administration of APOSEC at doses of secretome supernatant of 250 and 500 U/kg b.w. influenced 17 of 40 neuropharmacological parameters 1 hour after administration in a dose- related but transient way. All changes were no longer detectable after another 3 hours. At high dosages, and in view of the complex composition of potentially active ingredients such as cytokines and growth factors, some parameters, particularly motor activity and related parameters, may be impaired. Nevertheless, no behavioural effects have been observed in any of the *in vivo* toxicity studies already performed in mice, rats, and minipigs. APOSEC was not associated with any skin-sensitising properties in the LLNA in mice. The i.v. route of administration was used in a single-dose toxicity study in rats and in a 4-week i.v. toxicity study in mice to assess systemic and potential target organ toxicity. APOSEC is intended for topical use mixed with a hydrogel (NuGel); however, to mimic clinical use in DFU, the s.c. route of administration was considered more relevant. For this reason, a 4-week toxicity study was performed with s.c. administration in minipigs.

No clinical findings or signs of systemic intolerance reactions were observed in the acute toxicity study after single i.v. administration of APOSEC to rats (50, 250, and 500 U/kg b.w.). No changes in behaviour were noted, either. Body weight, body weight gain, and haematological, biochemical, and urinalysis parameters were not affected, and no macroscopic changes were observed at necropsy.

In the two pivotal 4-week repeated-dose toxicity studies conducted via the i.v. route in mice and the s.c. route in minipigs, the dosing regimen and study duration covered the intended clinical maximum use in patients. No test item–related clinical symptoms or pathological changes were detected in the animals. Based on data available from pharmacological studies, the lowest efficacious dose is estimated to be 1.88 U/cm^2^ in mice and 3.75 cm^2^ in pigs (2.5 U/1.237 cm^2^ wound; 125 U/kg in mice; 150 U/40 cm^2^ wound; 5 U/kg in pigs, respectively). The minimal dose for the treatment of DFU wounds currently proposed in the draft Phase IIa protocol is 25 U/wound/treatment, equivalent to 0.42 U/kg b.w. and 4.17 U/cm^2^. The planned maximum clinical dose for the treatment of DFU wounds is 200 U/wound/treatment, equivalent to 3.3 U/kg body weight and 100 U/cm^2^. For the final protocol, the safety factor and dose levels to be used in a Phase II clinical study will take into consideration the NOEL determined from the 4-week toxicity studies in mice (i.v.) and minipigs (s.c.). In selecting appropriate doses in toxicology studies, the proposed starting and maximum clinical doses of 0.42 U/kg and 3.3 U/kg were considered. These dose levels are well above the proposed clinical starting dose.

In conclusion, the results of these non-clinical investigations demonstrate the safety of APOSEC in different animal models. However, it must be noted that any extrapolation of the results to humans requires caution. Non-clinical animal studies are necessary per guidelines, and the animal species used have demonstrated great utility for the types of studies performed. In all of these studies, APOSEC was well tolerated, with no adverse events and no influence on circulatory, respiratory, or haematological parameters. APOSEC showed a good safety profile, and the non-clinical study findings reported here are adequate to support administration of APOSEC in Phase II clinical studies.

## Data Availability

Materials, data and associated protocols will be made available to readers upon request within material transfer agreements.
